# Author Index for Volume 100

**DOI:** 10.1038/sj.bjc.6605132

**Published:** 2009-06-09

Aaltonen, K 1055 Aaltonen, LA 511 Aavikko, M 1336 Abdi, E 1245 Abello, J 1755 Abnet, CC 551 Acevedo-Martinez, S 1073 Achen, MG 1784 Ackland, L 134 Adami, HO 1794 Adams, RA 251 Addou, S 1832 Afonso, L 487 Agarwal, R 305 Agarwal, S 941 Agostini, C 601 Ahmed, N 134 Ahn, JS 894 Aikou, T 153 Aithal, GP 178 Aittomäki, K 1055 Akhter, M 1812 Akiba, S 848 Al Olama, AA 426 Al Saati, T 1755 Alaiya, AA 1303 Alaoui-Jamali, MA 633 Al-Batran, SE 44 Aldaoud, A 44 Aleba, A 601 Alessandroni, P 881 Alexopoulou, DK 1659 Alfaro, C 1111 Alinger, B 1434 Allan, L 1867 Allen, NE 1817 Allum, W 1725 Almaguel, F 1073 Almeida, R 82 Alonzi, R 644 Altena, R 1861 Altés, A 1368 Altman, DG 1219 Aman, P 1687 Ambroisine, L 888 Ambros, IM 1471 Ambros, PF 1471 Ambrosone, CB 1680 Amini, R-M 1055 Amiot, M 366 Amoroso, D 1720 Amr, S 817 Anders, M 352 Andersen, CL 511 Anderson, AR 412 Anderson, JH 701, 1236 Anderson, LA 822 Anderson, LR 123 Anderson, M 1367 Anderson, N 923 Andersson, S 1303 Andersson, S-O 170 Ando, M 50 Andrèn, O 170 Andreuccetti, M 1720 Andrews, EJ 1589 Andrews, L 583 Ang, J 1373 Ang, JE 671 Anton-Culver, H 412, 524 Antonini, NF 266 Antonuzzo, A 1720 Anttila, S 1336 Aoyagi, K 389 Apicella, C 583 Apolone, G 1566 Appleby, PN 1817 Arcusa, A 442 Ardanaz, E 1817 Ardern-Jones, A 376 Argilés, JM 311, 713, 1014, 1211 Arioka, H 50 Arkenau, H-T 1373 Arts, K 311, 713, 1014, 1211 Ashcroft, M 1515 Ashley, DM 1012 Ashley, S 305, 1373, 1725 Aspe, JR 1073 Asumen, MG 1073 Auer, G 1303 Auranen, A 1315 Autier, P 174 Azuara, D 1534 Azzato, EM 1806

Baba, H 1937 Baier, P 360 Baiget, M 1368 Baker, L 1687, 1867 Balanathan, P 1784 Balasubramanian, R 1879 Balbi, C 1608 Baldelli, AM 881 Baldwin, KA 1465 Ballarini, M 28 Balsitis, M 1530 Banerji, U 1373 Bang, Y-J 298, 732 Bangma, C 13 Banovich, F 259 Bapat, B 1966 Baracos, VE 1581 Baranov, V 1603 Barboro, P 1608 Barile, C 1549 Barker, CL 1558 Barlow, M 684 Barnadas, A 442, 1368 Barr, R 1026 Barrett-Lee, PJ 684 Barriuso, J 1373 Barros-Silva, JD 487 Barry, BD 1452 Barter, S 1873 Barthel, R 1755 Bassi, C 246 Bataille, R 366 Batra, S 1425 Baumgarten, M 817 Beau-Faller, M 985 Becker, S 1303 Beckers, J 656 Beesley, AH 1926 Beesley, J 412 Beiske, K 1627 Beith, J 1245, 1250 Belien, JAM 145 Bell, BA 789 Bell, D 1068 Bell, JL 96 Bellù, F 840 Belohlavek, C 322 Benlimame, N 633 Bennett, RL 1043 Benoit, G 608 Benson, M 1725 Bento, MJ 487 Beral, V 538 Berchuck, A 412 Berg, JP 450 Bergqvist, M 334 Bergstrom, S 334 Berkers, L-M 1240 Berndt, A 623 Berndt, SI 822 Berney, DM 888 Bernhard, H 44 Berns, EMJJ 971 Bernstein, L 524, 1483 Berridge, GLC 206 Berrino, F 1817 Berton, A 1444 Berton Rigaud, D 315 Bertrand, R 1896 Bertuccio, P 558 Bhalla, K 1523 Bhasin, D 106 Biankin, AV 123 Biasco, G 1017 Bibeau, F 1330 Bielawski, J 626 Bihrmann, K 1205 Bingham, S 1817 Birch, JM 188 Birgisson, H 1540 Birnbaum, D 1755 Bisonni, R 881 Bjartell, A 1540, 1799 Bjøro, T 455 Blaakaer, J 993 Blagden, S 707 Blanchard, F 1330 Blanks, RG 1043, 1832 Blomqvist, C 811, 1055 Blows, F 1806 Blum, HE 360 Boccardo, F 1608 Boccardo, S 1608 Bodman-Smith, M 1697 Boegl, M 1365 Boeing, H 1817 Bohlken, A 96 Boissière, F 1330 Bokaee, S 1889 Bolzonella, C 1549 Bonanomi, AG 259 Bonilla, M 1026 Boniol, M 174 Bonnard, C 1358 Bononi, A 1549 Bootle, D 315 Bordoni, A 1087 Bortoli, A 259 Bosch, FX 1191 Bosetti, C 558 Bougeard, G 1330 Bourbouloux, E 315 Bourré, L 723 Bouvier, V 1230 Boyd, AW 1095 Brandão, C 487 Brandi, G 1017 Brannan, RA 1393 Breakefield, XO 1603 Brenner, H 858 Brenton, JD 1873 Brewster, W 412 Brimmell, M 1347 Britton, P 1873 Brodeur, GM 1471 Brooks, A 707 Brouwer, CAJ 1861 Brown, G 1725 Brown, J 701 Brown, L 113 Brown, R 758 Brown, SBF 680, 807 Brundler, M-A 1292 Brunetti, IM 1720 Büchler, MW 246 Budroni, M 840 Bueno-de-Mesquita, HB 1817 Buffler, PA 863 Bugalho, MJ 1777 Bull, D 538 Bunch, KJ 213 Burchell, JM 1746 Burchill, SA 1627 Burger, H 228 Burger, M 1949 Burger, MPM 913 Burkart, C 44 Burniat, A 1013 Bursi, S 1720 Butt, AJ 123 Buxton, ILO 1465 Byrski, T 1508 Bziouech, H 1755

Caldas, C 1517, 1873 Caldas, H 106 Calderan, L 1575 Calhau, C 1120 Callam, M 598 Calvo, A 932 Calvo, F 315 Cambien, B 1755 Cameron-Smith, D 134 Campana, F 1048 Campbell, C 1852 Campbell, FM 807 Campo, L 405 Campone, M 315 Camponovo, A 1087 Canaani, D 1213 Candiloro, I 405 Caniza, M 1026 Canney, PA 684 Cannon, SR 1406 Cantwell, MM 174, 1492 Cao, P 1267 Capellà, G 1534 Caporaso, NE 1806 Caraceni, A 1566 Card, TR 178 Cardwell, C 1492 Carew, JS 1523 Carlsen, NLT 853, 1212 Carmignani, G 1608 Carneiro, F 487 Carney, ME 412 Carter, PJ 1367 Casadomé, L 1534 Caspari, C 590 Castelao, JE 834 Castellsagué, X 1191 Castiglione, G 259 Catalano, V 881 Catena, R 932 Catney, D 174 Catusse, J 1949 Cavaco, BM 1777 Cazzola, L 259 Cejka, D 1739 Cen, L 106 Chamoto, K 1135 Chan, ATC 663 Chan, C 106 Chang, CJ 1765 Chang, ET 524 Chang, K-J 563 Chang, K-P 1002 Chang, M-C 1144 Chang, S-H 865 Chang, Y-S 1002 Chang-Claude, J 1680 Chaouachi, K 1015 Chapman, P 167 Chappell, E 1966 Charbonnier, F 1330 Chatenoud, L 558 Chau, I 1704, 1725 Chekerov, R 1731 Chen, C 412 Chen, C-A 1144 Chen, G-H 1002 Chen, H-C 1002 Chen, K-M 563 Chen, MF 1765 Chen, S 1912 Chen, S-J 1002 Chen, TH-H 563 Chen, X 412 Chen, Y-H 1002 Chen, Y-L 1144 Chenevix-Trench, G 412 Cheng, W-F 1144 Cheung, BB 96 Cheung, IY 1627 Chiang, Y-C 1144 Chiara, S 1720 Chirlaque, M-D 1817 Choe, K-J 732 Choi, DH 894 Choi, IS 298 Chomienne, C 918 Chong, PY 676 Chou, W-Y 578 Chou, Y-C 578 Choudhury, B 370 Christensen, JG 941 Christensen, LL 511 Christensen, S 1503 Christians, U 923 Christmann, M 322 Chu, C-H 578 Chua, S 305 Ciatto, S 259 Cid, MO 1777 Cisek, K 106 Clamp, AR 1 Clark, SJ 1534 Clarke, AR 221 Clarke, C 524 Clausen, H 1746 Clifford, G 532 Closset, J 1444 Clyne, M 376 Cnattingius, S 803 Coebergh, JWW 77, 901 Coffey, JC 1452 Cohn, SL 1471, 1627 Colditz, GA 611 Coleman, DA 1832 Coleman, LJ 1393 Coley, HM 571 Collins, DJ 644 Collins, TL 1755 Colomiere, M 134 Confortini, M 259 Contegno, F 28 Conti, DV 834 Conti, M 608 Cooke, TG 680, 807 Coombes, RC 160, 1061, 1879 Cooper, CS 240, 888 Copercini, M 1771 Copier, J 1697 Corey, E 1068 Corli, O 1566 Corrie, PG 1245 Cortés, A 1368 Cortessis, VK 834 Corvilain, B 1013 Cotter, TG 1452 Coumbos, A 1731 Coupland, VH 167, 527 Coyle, B 1292 Cozen, W 524 Craig, GT 1128 Cramer, DW 412 Cramer, T 772 Crea, P 123, 405 Crepaldi, G 1549 Cretin, J 601 Crippa, S 1087 Croci, D 28 Crook, T 571, 1687 Crozier, JEM 701 Culhane, AC 1452 Cunningham, D 1704, 1725 Cunningham, JC 412 Cupini, S 1720 Cutuli, B 1048 Cuzick, J 888 Cvancarova, M 455 Cybulski, C 1508 Czene, K 1358, 1486

Daducci, A 1575 Daftary, D 1966 Dahse, J 623 Dahse, R 623 Dai, M 532 Daidone, MG 739 Daito, M 494 Dal Maso, L 840 Dalgleish, A 1697 Daly, MB 583 Danjoh, I 389 Danos, O 1018 Darabi, H 1358 d′Arcy, JA 644 Darnton, A 1175 Darrington, RS 1165 Daskalow, K 772 Davidson, BR 617 Davidson, SE 1558 Davidson, WF 817 Davies, EA 167, 545 Davies, H 370 Davies, J 1889 Davies, M 1824 Davis, ID 1250 Day, JM 476 de Beaux, AC 63 de Bono, JS 671 De Dosso, S 1087 de Haas, EC 1861 de Kok, IMCM 1240 de Lafontan, B 1048 De Leon, M 1073 De Lisi, V 840 de Martel, C 194 de Pinieux, G 918 de Rosa, F 1017 de Sanjose, S 1191 de Souza, PL 649 De Vuyst, H 532 de Winton, E 693 de Wit, R 13 Deandrea, S 1566 DeAngelis, S 106 Deans, DAC 63 Deapen, D 524 Debniak, T 1508 De-Bono, J 1373 Decaestecker, C 1444 Decaudin, D 918 Degrassi, A 1575 del Río, E 1368 Demetter, P 1444 D'Emidio, S 881 Descamps, G 366 Devière, J 1444 Dewar, J 1867 Dhillon, A 617 Di Donato, S 1720 Di Fiore, F 1330 Diamandis, EP 1659 Diament, RH 1530 Diaz, M 1191 Diaz, R 932 Diaz Miqueli, A 950 Diaz-Gonzalez, JA 932 DiCioccio, RA 412, 993 Dieleman, LA 1581 Dietrich, P-Y 1771 Diez, S 1165 DiGiovanna, MP 941 Dilis, V 1817 Dilulio, J 37 Ding, J 494 Dinis-Ribeiro, M 487 Dirix, LY 971, 1277 Diss, T 1406 Ditsch, N 590 Dix, D 82 Dobrovic, A 1012 Doecke, JD 795, 1095, 1514 Doherty, JA 412, 993 Dohollou, N 601 Doi, R 246 Doki, Y 1647 Dolan, OM 174 Dollinger, MM 1032 Domingues, R 1777 Donato, A 840 Donnelly, D 174 Dono, K 1647 Donovan, J 426, 1198 Donovan, M 1452 dos Santos Silva, I 1824 Douard, R 608 Doughty, JC 680 Dowdy, S 89 Draper, GJ 213 Dreef, EJ 1617 Driemel, O 623 Driver, KE 1806 Droupy, S 608 Drummond, E 693 Drummond, KJ 1012 Duan, L 1483 Dubrot, J 1111 Dudek, AZ 1379 Duffy, SW 220, 1198, 1873 Duijm, LEM 77, 901 Dumont, J-E 1013 Duniho, S 1367 Durand, L 918 Dutreix, C 315 Dvorak, AM 865 Dvorak, HF 865 Dyson, PJ 1061 Dzolganovski, B 82

Eastham, J 888 Easton, DF 426, 993 Ebert, M 352 Eckhardt, SG 923 Eden, TOB 188 Eder, W 1434 Edkins, S 370 Edlund, CK 412 Edwards, J 680, 807 Edwards, R 412 Edwards, S 426 Eeles, R 376, 426 Egashira, N 764 Eggleston, IM 723 Einarsdóttir, K 1358 Ekbom, A 1794 Eklund, CM 1846 Ekman, S 334 Eljamel, S 1867 Elsberger, B 807 El-Sheikh, S 959 Elst, HJ 1277 Enderling, H 1917 Endlicher, E 1032 Engebjerg, MC 200 Engels, EA 817, 822 Engelstaedter, V 360 Erro, L 1111 Eschwège, P 608 Eskens, FALM 228 Essink-Bot, M-L 70 Evans, WK 56 Eward, KL 959 Eyholzer, M 1343 Eyre, R 188

Faber, J 713, 1211 Falcini, F 840 Falcone, A 881, 1720 Fall, K 170 Falzon, M 959 Farace, P 1575 Farnell, DJJ 1558 Fay, M 693 Fearon, KCH 63 Fedeli, SL 881 Federico, M 840 Feichtinger, R 1434 Feltbower, RG 188 Fennelly, D 381 Ferrari, N 1608 Ferrazzi, E 1549 Ferreira, P 487 Ferrero, JM 601 Ferretti, S 840 Fichtner, I 950 Field, CJ 1581 Fietkau, R 291 Filipović, A 1879 Finch, A 421 Findlay, J 134 Finn, P 758 Firth, MJ 1926 Fisher, D 251 Fisher, G 888 Flaherty, KT 431 Flanagan, AM 1406 Fleuren, GJ 1617 Floor, K 145 Folini, M 739 Folprecht, G 44 Fondrinier, E 1048 Ford, J 1926 Forest, V 1896 Fornaro, L 881, 1720 Forouhi, P 1873 Fos, J 1343 Fosså, A 455 Fosså, SD 455 Foster, CS 240, 888 Foster, PA 476 Foulis, AK 701 Fourquet, A 1048 Fox, C 174 Fox, SB 405 Fracheboud, J 901 Fragoso, M 487 Franc, B 1013 Franceschi, S 194, 532, 840, 1191 Franklin, M 1379 Fraser, J 1315 Frattini, M 1087 Frebourg, T 1330 Frederiksen, K 185 Freedman, ND 551 Freitas, JR 1926 Fric, D 601 Friedman, HS 1012 Frigo, AC 1549 Friis, S 200, 1503 Frincke, J 1068 Fritzer-Szekeres, M 1739 Fuchs, M 1032 Fueger, BJ 1739 Fuentes, SL 1026 Fuereder, T 1739 Fuh, B 106 Fujiwara, Y 50 Fukagawa, T 153, 1937 Fukaya, M 389 Fung, MC 118 Fusco, M 840 Futreal, PA 370

Gäbele, E 1032 Gaber, A 1540 Gabra, H 707 Gadalla, S 822 Gadalla, SM 817 Gago-Dominguez, M 834 Gala, J-L 608 Galettis, PT 649 Galiè, M 1575 Galitovskiy, V 412 Galleges Ruiz, MI 145 Gallinger, S 1966 Galustian, C 1697 Gan, HK 1012 Gandellini, P 739 Gangadharan, P 848 Ganju, V 37 Gao, H 1957 García-Echeverría, C 1267 Garmo, H 170, 811 Garrett, C 1842 Garsd, A 1068 Gaskarth, M 1873 Gatter, KC 405 Gaub, MP 985 Gavin, AT 174 Gayther, SA 412, 993 Geay, J-F 601 Gedda, L 334 Geng, H 663 Gentry-Maharaj, A 412, 993 George, S 24 Gerald, W 888 Gerber, H-P 113 Ghaneh, P 246 Giaccone, G 145 Giacomin, A 840 Giard, S 1048 Gibbs, J 1842 Gietema, JA 1861 Gikas, P 1406 Gil, M 442 Giles, FJ 1523 Gilham, C 1175 Gilmour, IM 684 Giordani, P 881 Giovannetti, E 1120 Giuntini, F 723 Giustini, L 881 Glen, J 598 Glimelius, B 1540 Glynne-Jones, R 1666 Goedert, JJ 817 Goh, HK 663 Gohda, K 494 Goldhirsch, A 28 Gomez-Bougie, P 366 Gondos, A 858 Gonzalez, A 1111 Gonzalez, C 1817 González, S 442 Gonzalez-Moreno, O 932 Goode, EL 412 Goodman, MT 412 Goossens, A 913 Gopalan, A 888 Gordon, KA 113 Gores, GJ 1385 Gorselink, M 311, 713, 1014, 1211 Gorski, B 1508 Gorter, A 1617 Goto, M 344 Götze, H 908 Gøtzsche, PC 219 Gout, I 1406 Gouttebel, MC 601 Grabenbauer, GG 291 Graham, J 707 Grainge, MJ 178 Grande-Pulido, E 1111 Grant, RM 1867 Graziano, F 881 Grazzini, G 259 Greaves, M 863 Greco, MT 1566 Green, AC 795, 1514 Green, J 538 Greenberg, D 1198, 1806 Greenberg, ML 1026 Greenman, C 370 Greeno, EW 1379 Greenwood, CM 1966 Grell, K 185 Greten, TF 19 Gretschel, S 352 Grewal, IS 113 Griffiths, JR 789 Grillo, F 617 Groenewoud, JH 901 Gronwald, J 1508 Groshen, S 834 Grünberg, K 145 Grundy, RG 1292 Grunfeld, E 56 Guenther, T 1666 Guérin, E 985 Guittet, L 1230 Gullberg, B 1799 Gullbo, J 334 Gupta, S 1026 Gurpide, A 1111 Gusella, M 1549 Guy, M 426

Haagsman, HP 311, 1014 Haase, W 1680 Habbema, JDF 1103, 1240 Haber, M 96, 1471 Haddad-Guichard, Z 601 Hadjeres, R 1896 Hahnfeldt, P 1917 Hall, A 426 Hall, P 1358, 1486, 1794 Hall, PS 1513 Hallmans, G 1817 Hamdy, F 426, 1198 Hamel, E 932 Han, C 405 Han, S-W 298 Han, Y 1154 Hanby, AM 1393 Hansson, MG 8 Harada, H 747 Harder, J 360, 1032 Hardiman, P 1824 Hardy-Bessard, AC 601 Härkki, P 1315 Harrington, KJ 470, 1889 Harris, AL 405, 1666 Harris, LN 941 Hartmann, TN 1949 Harvey, R 979 Hasegawa, H 1943 Hasegawa, R 870 Hatch, J 1175 Hatmal, M 89 Hauss, J 908 Hawkins, CJ 1012 Hayashi, T 501 Haydon, A 1250 Haylock, RGE 206 Hayward, O 1250 Hazelbag, S 1617 He, Q 633 Hedde, JP 291 Hedley, DW 1267 Heikkilä, P 1055 Helleman, J 971 Hellman, K 1303 Hellman, U 1303 Hellström, A-C 1303 Helmbold, I 1680 Hemminki, K 829, 1499 Henderson, K 524 Henshall, SM 123 Heo, DS 732 Herath, NI 1095 Heriot, AG 693 Hernandez-Suarez, G 1184 Hernes, E 450 Herrero, R 1191 Hersey, P 1250 Hervas-Stubbs, S 1111 Hesselius, P 334 Hestvik, UE 450 Hicks, RJ 693 Higashi, M 344 Higashino, F 1943 Higashiyama, S 1320 Higgins, DG 1452 Hiissa, J 1315 Hinke, A 1032 Hinz, A 908 Hirakawa, A 50 Hiraoka, M 747 Hirata, T 50 Hirata, Y 1320 Hirohashi, S 1257 Hirotsu, M 1957 Hiyama, E 1627 Hlatky, L 1917 Ho, YT 476 Höcker, M 352, 772 Hodgson, J 1175 Hoffmann, E 376 Hoffmann, M 44 Hogdall, CK 412, 993 Høgdall, E 412, 993 Hogg, A 693 Hol, L 1103 Hollenbeck, AR 551 Hollerbach, C 1032 Hollerbach, S 1032 Holmberg, L 811 Holt, C 959 Holt, SK 412 Holte, H 455 Honarpisheh, H 1393 Hoogerbrugge, N 266 Hopper, JL 583 Horgan, PG 701, 1236 Horlock, C 1061 Horsch, M 656 Horsman, D 421 Horvath, LG 1784 Hoshi, S-L 281 Hoskin, PJ 644 Hosoi, H 399 Hotakainen, K 1540 Houlston, RS 233, 1674 Houston, JG 1867 Howard, SC 1026 Howe, FA 789 Hrabé de Angelis, M 656 Hsieh, CC 1794 Hsieh, C-Y 1144 Hsieh, SY 1765 Hsu, G-C 578 Hu, Y-H 1144 Huang, C-Y 1144 Huang, P 1912 Hubner, RA 233 Huebner, G 44 Huget, P 1277 Hughes, TA 1393 Huh, SJ 894 Huhtinen, K 1315 Humphreys, K 1358 Hunter, N 213 Hurme, M 1846 Hutzen, B 106 Huvila, J 1315 Huzarski, T 1508 Hyde, EI 1903 Hyland, J 381

Iacopetta, B 676 Idowu, B 1406 Iehara, T 399 Ikeda, M 870 Ikeda, S 1400 Iliadou, A 803 Im, S-A 298, 732 Im, Y-H 894 Imai, M 464 Inokuchi, M 782 Inoue, M 181 Intrieri, T 840 Introini, C 1608 Ishihara, H 494 Ishii, H 1937 Ishikawa, K 153 Ismail, M 1889 Isohata, N 389 Itasaka, S 747 Ito, S 1937 Ito, Y 676 Itoh, J 764 Iversen, ES 412 Iwasaki, M 181, 1812 Iwatsuki, M 1937 Iyer, R 1842

Jaakkola, PM 874 Jack, RH 545 Jacobs, IJ 993 Jaffey, J 56 Jahn, A 44 Jalkanen, J 1315 James, PW 188 Jansen, G 1120 Jansen, H 713, 1211 Jarrom, D 1903 Javle, M 1842 Jayalekshmi, PA 848 Jayatilleke, N 24 Jayson, GC 1 Jean-François, R 1896 Jenab, M 1817 Jenkinson, HC 188 Jennison, E 412 Jensen, AØ 200 Jensen, M-B 1205 Ji, J 829, 1499 Ji, SH 894 Jiang, X 834 Jin, L 1013 Jirström, K 1540 Joh, T 1320 Johansen, C 185 Johansson, J-E 170 Johansson, M 1540 Johns, TG 1012 Johnsen, NF 1817 Johnson, M 1837 Johnson, MG 1755 Johnston, S 305 Jonas, M 113, 1367 Jones, AL 274, 684 Jones, M 89 Jones, ND 1434 Jones, RL 305 Jöns, T 772 Jonsson, H 220 Jørgensen, KJ 219 Joseph, D 676 Josiah, D 106 Jöst, E 322 Judson, I 1373 Jugurnauth, S 426 Julien, S 1746 Junnila, J 1315

Kaaks, R 1817 Kahlert, S 590 Kaina, B 322 Kaiser, U 44 Kakiuchi, S 501 Kakuguchi, W 1943 Kamangar, F 551, 1016 Kamiya, T 1320 Kang, WK 894 Kaniwa, N 870 Kaplan, RS 251 Karagas, MR 200 Karimdjee, BF 1755 Karjalainen, A 1336 Kasai, S 1257 Kasamatsu, T 1400 Kataoka, H 1320 Kathmann, I 1120 Kato, T 1400 Katsumata, N 50 Kattan, MW 888 Kawano, T 782 Kawano, Y 1165 Kay, E 251 Kaye, AH 1012 Kaye, S 1373 Keane, M 1415 Keeney, GL 89 Kees, UR 1926 Kefford, R 1250 Kefford, RF 1245 Kegler, D 311, 713, 1014, 1211 Kelly, MA 1452 Kemmner, W 352, 772 Kendall, GM 213 Kenny, S 251 Kenter, GG 1617 Kerkmeijer, LG 979 Keshtgar, MR 1492 Kettunen, E 1336 Key, TJ 1817 Khamly, K 37 Khan, J 1471 Khan, S 1073 Khaw, K-T 1817 Khramtsov, A 1154 Killeen, SD 1589 Kim, D-W 732 Kim, J-S 732 Kim, M-A 298, 732 Kim, NK 732 Kim, SJ 494 Kim, SR 870 Kim, TM 732 Kim, T-Y 298, 732 Kim, WH 298, 732 Kim-Sing, C 421 King, M 274 Kingsbury, SR 959 Kirk, GD 799 Kirwan, WO 1452 Kiserud, CE 455 Kita, Y 153 Kitagawa, Y 1943 Kitamura, H 1135 Kitamura, T 1943 Kjaer, SK 412 Klaassen, RJ 82 Klassen, A 82 Klawitter, J 923, 923 Kneebone, R 24 Knight, JA 1966 Knops, AM 913 Ko, Y 291 Koach, J 96 Kobayashi, S 1647 Kocher, M 291 Koehm, J 590 Kofler, B 1434 Kohne, CH 44 Kojima, K 782 Kokubu, A 1257 Kolmakova, J 941 Komiya, S 1957 Kondo, M 281 Kondo, S 870 Konecny, GE 89 Koopman, M 266 Korangy, F 19 Korbelik, M 626 Korkeila, E 874 Kortman, GAM 266 Kosaka, Y 153 Kosmehl, H 623 Kotasek, D 1245 Kote-Jarai, Z 426 Kouno, T 50 Kratzke, RA 1379 Krauß, O 908 Kretzschmar, A 44 Kromeyer-Hauschild, K 623 Kropp, S 1680 Krüger-Kjaer, S 993 Kruhøffer, M 511 Kubota, E 1320 Kudahetti, S 888 Kudo, E 501 Kuehn, W 1731 Kuhnt, S 908 Kuipers, EJ 70, 1103 Kujari, H 1315 Kulinskaya, E 160 Kullmann, F 44, 1032 Kumagai, J 782 Kumar, D 1697 Kumar, P 1379 Kumaran, GC 1 Kunitoh, H 464, 1037 Kuntner, C 1739 Kuo, W-H 563 Kuosma, E 1336 Kuppen, PJ 494 Kurahashi, N 181 Kuroda, Y 1438 Kuroshima, T 1943 Kurtz, DM 1385 Kusakabe, T 764 Kuvshinoff, B 1842 Kypta, RM 1165 Kyrgidis, A 670

La Vecchia, C 558 Lagiou, P 1794 Lamy, A 1330 Lancrenon, S 1048 Landgren, O 822 Langenberg, P 817 Langer, O 1739 Langridge, WHR 1073 Larranaga, N 1817 Larsson, H 853, 1212 Lassmann, S 360 Laudico, A 858 Launoy, G 1230 Laurberg, S 511 Lavaux, T 985 Laviano, A 311, 713, 1014, 1211 Law, C-L 113 Le Pessot, F 1330 Leathem, AJ 1492 Lecomte, N 918 Lee, CH 1765 Lee, I-M 611 Lee, J-I 894 Lee, KH 298 Lee, K-U 732 Lee, K-W 298 Lee, MH 298 Lee, N-S 298 Lee, P-H 563 Lee, S-H 732 Leese, MP 476 Leggett, BA 1095 Legrain, M 985 Lehmann, A 908 Leibfritz, D 923 Leick, M 1949 Leitão, D 487 Leitch, EF 1236 Leite, V 1777 Leitzmann, MF 551 Lemanski, C 1048 Lemm, M 950 Lemos, C 1120 Lennartsson, J 334 Lennerz, V 322 Leong, T 37, 693 Leongamornlert, DA 426 Leppard, B 24 Lesniak, MS 1154 Lesniewski-Kmak, K 1379 Levack, P 1867 Levene, A 617 Levillain, R 1230 Lévy, E 601 Levy, V 315 Li, C 106 Li, L 1659 Li, L-K 532 Li, N 532 Li, P-K 106 Li, X 829 Li, Y 1358 Li, YQ 1358 Liao, W-L 1002 Lichtenegger, W 1731 Liebmann, A 908 Ligtenberg, MJL 266 Lilla, C 1680 Lim, DH 894 Limina, RM 840 Lin, J 106 Lin, L 106 Lin, YJ 1765 Lindblom, A 1674 Lindner, D 1287 Link, H 44 Linklater, KM 167 Linseisen, J 1817 Lipworth, L 1794 Lise, M 840 Little, P 24 Little, V 1824 Litwin, A 1842 Liu, B 532 Liu, C-Y 1002 Liu, ET 1358 Liu, J 1358 Lobitz, S 772 Loddo, M 959 Lomnytska, M 1303 London, WB 1471 Lopergolo, A 739 Lopes, C 487 Lopez, J 571 Lopez-Picazo, JM 1111 Loric, S 608 Lorigan, P 1250 Loubeyre, P 1771 Louie, KS 1191 Loupakis, F 881, 1720 Louwman, MWJ 77, 901 Lovejoy, DB 96 Lovric, MM 1012 Low, YL 1358 Lowe, J 1292 Lowy, A 24 Lu, Y 524 Lubinski, J 1508 Lucenteforte, E 558 Luiking, Y 713, 1211 Lundberg, F 803 Lurie, G 412 Lynge, E 220, 1205

Ma, H 524 MacKay, J 693 MacLean, AB 1824 MacRobert, AJ 723 Madeddu, A 840 Madi, A 251 Maeda, N 1438 Mæhlen, J 219 Maes, H 1277 Mahalingam, D 1415, 1523 Mahoney, CL 370 Maira, S-M 1267 Mäkinen, J 1315 Malek, NP 19 Malekzadeh, R 1016 Mallon, EA 680, 807 Mandelzweig, L 1021 Mangone, L 840 Manivet, P 608 Manno, M 1966 Manns, MP 19 Mantellini, P 259 Mapua, C 858 Marangoni, E 918 Maraveyas, A 1837 Marcuello, E 1368 Maréchal, R 1444 Margelí-Vila, M 442 Margison, GP 1245, 1250 Mari, D 881 Mariani, A 89 Marinelli, R 1549 Mariotte, N 1230 Maris, JM 1471 Marshall, C 598 Marshall, GM 96 Marsiglia, H 1048 Martin, RM 1198 Martin, V 1087 Martini, V 1755 Marubashi, S 1647 Marzola, P 1575 Mashima, T 1369 Masi, G 1720 Massard, G 985 Massuger, LF 979 Matera, A 37 Matsubara, K 1647 Matsumoto, K 50 Matsunoshita, Y 1957 Matsushima, T 494 Matsushita, M 1438 Matsuura, N 1647 Matthay, KK 1627 Matuszewski, M 1508 Maughan, TS 251 Maxwell, F 701 Maxwell, JA 1012 Maxwell, PH 1515 Maxwell, RJ 644 Mayer, D 1824 Mayor, R 1534 Mayr, JA 1434 Maziak, DE 56 Mazzucchelli, L 1087 McArthur, G 1250 McCabe, C 1513 McCarthy, O 274 McCormack, RT 160 McCormack, VA 1824 McDonald, PJ 1666 McElwaine, JN 1393 McGlynn, LM 807 McGown, G 1250 McGregor, JR 1530 McGuire, V 412, 993 McIntosh, HM 1852 McKee, RF 701, 1236 McKee, TA 1771 McKinney, PA 188 McLaughlin, JK 1503 McLeay, T 1867 McMillan, DC 701, 1236 McNally, RJQ 188 McNeil, CM 123, 405 McPherson, RAC 649 Meade, AM 251 Medina, JC 1755 Meerding, WJ 1240 Mehmet, H 1415 Mei, T 1912 Meijer, C 1184 Meijer, CJ 1191 Meijer, GA 145 Mekenkamp, L 266 Melero, I 1111 Méndez, F 1184 Menges, M 44 Menon, D 1549 Menon, U 412, 993 Merchant, S 626 Merigo, F 1575 Mery, E 1048 Messmann, H 1032 Metcalfe, KA 421 Meunier, A 1048 Meyer, B 1697 Meyer, T 1975 Michael, M 37 Michel, P 1330 Middleton, MR 1245, 1250 Middleton, RJ 174 Mikeska, T 1012 Miles, D 1746 Millar, EKA 123, 405 Miller, S 1292 Millet, MA 1755 Milner, AD 37, 693 Mimori, K 153, 1937 Mincheva-Nilsson, L 1603 Minn, H 874 Minucci, S 28 Miot, F 1013 Mirasol-Lumague, MR 858 Mironenko, T 370 Misawa, A 399 Mitchell, H 707, 979 Mizokami, M 181 Mizoshita, T 1320 Mizushima, T 1320 Molano, M 1184 Molinari, F 1087 Møller, H 167, 527, 545, 888 Monden, M 1647 Montanari, M 1566 Montero, E 950 Moore, M 246 Moore, PA 1367 Moorman, PG 412 Morash, C 56 Moreau, P 366 Moreno, A 442 Moreno, V 1184, 1534 Morey, AL 123 Morgan, A 436 Morgan, R 470 Mori, K 764 Mori, M 153, 1647, 1937 Mori, Y 1320 Morinibu, A 747 Morishita, M 1061 Morizane, C 870 Morrison, J 426 Mortimer, P 1245, 1250 Moss, SM 1043, 1832 Moug, SJ 1530 Moura, MM 1777 Moutereau, S 608 Moyes, LH 1236 Moysich, KB 412, 993 Mrkonjic, M 1966 Mubwandarikwa, E 188 Mucci, L 170 Muderspach, L 89 Mueller, BU 1343 Mueller, M 1739 Muir, K 426 Muirhead, CR 206, 213 Mulcahy, H 381 Mulholland, S 426 Muller, A 37 Muller, RP 291 Muñoz, Á 1184 Muñoz, M 442 Muñoz, N 1184, 1191 Munro, AJ 1867 Munzone, E 28 Murakami, M 1647 Murayama, T 782 Murillo, R 1184 Murray, LJ 174 Musgrove, EA 123

Nagano, H 1647 Nagy, JA 865 Nagy, N 1444 Nair, RRK 848 Nakagawara, A 1471 Nakanishi, H 1937 Nakasono, M 501 Nakayama, S 1438 Nam, DH 894 Nambooze, S 799 Nandi, S 1154 Nannuru, KC 1638 Narod, SA 421, 1508 Nasrollahzadeh, D 1016 Natsugoe, S 153 Naumann, SC 322 Nawrocki, ST 1523 Nazareth, I 274 Neal, DE 426, 1198 Neal, RD 1852 Neale, RE 795, 1514 Near, AM 412 Negri, E 558, 1566 Neoptolemos, JP 246 Ness, RB 412, 993 Nesterova, A 1367 Neuhaus, T 291 Neuhausen, SL 524 Neuper, C 89 Neureiter, D 1434 Neuville, A 985 Nevanlinna, H 1055 Newman, SP 476 Ng, M 693 Ng, SS 676 Ngan, SY 37, 693 Nguewa, PA 932 Nicolato, E 1575 Nieto, J 1824 Nilsson, G 811 Nilsson, J 1603 Nishida, Y 344 Nishimura, T 1135 Nishio, M 464 Nishiwaki, Y 464 Nizar, S 1697 Noda, K 464 Noda, T 1647 Noguchi, S 494 Nokihara, H 1037 Nolè, F 28 Nomoto, M 344 Nordstrand, A 1603 Norris, M 96 Northover, JMA 1666 Nöthlings, U 1817 Numada, S 494 Nuver, J 1861 Nymark, P 1336

Oates, J 1725 O'Brien, L 426 O'Brien, M 305 Ocama, P 799 Ochiya, T 1257 O'Connor, D 1347 Oden-Gangloff, A 1330 O'Donoghue, D 381 Odumosu, O 1073 Offman, J 376 Oflazoglu, E 113 Ogasawara, N 1320 Ogretmen, B 626 Oh, D-Y 298 O'Hagan, JA 206, 213 Ohe, Y 1037 Ohkuri, T 1135 Ohta, A 389 Ohta, H 389 Ojima, H 1257 Okines, A 1725 Okulicz, W 1794 Okusaka, T 870 Olesen, AB 200 Olmos, D 671, 1373 Olschewski, M 360 Olsen, AH 1817, 1205 Olsen, JH 1503 Olsson, H 1799 Onda, T 1400 O'Neill, A 1873 Opio, CK 799 Opitz, OG 360 Opstad, KS 789 Orfeuvre, H 601 Orlotti, NI 739 Ørntoft, TF 511 O'Rourke, K 56 Osamura, YR 764 Oskay-Oezcelik, G 1731 O'Sullivan, J 381 O'Toole, SA 123, 405 Otremba, B 44 Otto, F 360 Oudet, P 985 Overton, C 1824 Overvad, K 1817 Owen, E 246

Pabst, T 1343 Packham, G 1347 Padbury, R 246 Padhani, AR 644 Padrini, R 1549 Palazón, A 1111 Palli, D 1817 Palmer, C 1245 Palmieri, C 160 Pamecha, V 617 Pande, A 1842 Pandey, ON 1379 Pandeya, N 795, 1514 Pandha, HS 470, 1889 Pantel, K 160 Paraiso, D 601 Paré, L 1368 Park, K 894 Park, MJ 894 Park, SR 298 Park, W 894 Park, YH 894 Parkes, S 188 Parsonnet, J 194 Pashayan, N 1198 Pasini, F 1549 Patel, D 56 Paterson-Brown, S 63 Payne, R 160 Peacock, SJ 583 Pearce, CL 412, 993 Pearce, MS 188 Pearson, ADJ 1471, 1627 Pedersen, BL 853 Pedersen, JS 1784 Peh, BK 676 Peinado, MA 1534 Pelicci, PG 28 Pelucchi, C 558 Penault-Llorca, F 1048 Pennati, M 739 Percharde, M 571 Perera, KU 1926 Perez, R 950 Perez-Gracia, JL 1111 Perheentupa, A 1315 Pesenti, E 1575 Peter, MB 1393 Peters, GJ 145, 1120 Petersen, BL 1212 Pethe, VV 1966 Peto, J 1175 Petrini, I 1720 Pezzella, F 405 Pfeiffer, M 1949 Pfeiffer, RM 817, 822 Pharoah, P 1198 Pharoah, PDP 412, 993, 1806 Phelan III, J 1842 Phesse, TJ 221 Phillipson, MA 206, 213 Picco, G 1746 Pierce, JS 626 Piesiak, W 1508 Piffer, S 840 Pike, MC 834, 993 Pinese, M 123 Pinto, AE 1777 Pioud-Martigny, R 1048 Pirianov, G 1415 Pirie, K 538 Pirinen, R 1336 Piselli, P 840 Pitfield, T 160 Plowright, L 470 Plumb, JA 758 Plummer, CJ 684 Plummer, M 194, 532 Pockney, P 24 Podratz, KC 89 Polder, JJ 1240 Polesel, J 840 Polinder, S 70 Poll, A 421 Polus, M 1444 Poma, P 739 Ponder, BAJ 993 Pontén, F 1540 Poon, FF 663 Popanda, O 1680 Posso, H 1184 Postel-Vinay, S 1373 Postma, A 1861 Potter, BVL 476 Poulsen, AH 1503 Poupon, M-F 918 Poutanen, M 1315 Praud, D 558 Presneau, N 1406 Press, MF 89 Preusser, M 1739 Prevost, T 959 Price, T 37 Prieto, L 442 Primrose, J 24 Prinz, C 1365 Pritchard, S 82 Proctor, I 959 Pross, M 352 Provencal, J 601 Pujade-Lauraine, É 601 Pulte, D 858 Punt, CJA 266 Puppo, P 1608 Purdie, C 1687 Purohit, A 476 Putter, H 494 Pyrhönen, S 874

Qian, ZR 501 Qiao, Y-L 532 Qiu, G-H 663 Quaye, L 993 Quinlan, P 1687 Quoix, E 985

Radovanovic, I 1020 Radulovic, M 376 Raghavan, D 1287 Rahman, MM 501 Rajan, B 848 Rake, C 1175 Ramus, SJ 412, 993 Ranson, M 1245, 1250 Rasheed, S 1666 Rashid, M 959 Rasul, S 1879 Rautenberg, TA 1513 Ray-Coquard, I 601 Raymond, E 315 Reading, C 1068 Rebolj, M 1240 Rechnitzer, C 853, 1212 Recktenwald, CV 656 Redaniel, MT 858 Redmond, HP 1452, 1589 Redrado, M 932 Reed, MJ 476 Reeves, GK 538 Regan, N 106 Rêgo, S 487 Reijerink, JCIY 1103 Repaci, E 1608 Reuter, V 888 Reynolds, P 524 Rha, SY 663 Ribeiro, R 1026 Riboli, E 1817 Ricci, S 1720 Richard-Fiardo, P 1755 Richardson, A 274 Richardson, DR 96 Riddell, A 1725 Riehle, D 89 Riethdorf, S 160 Riffkin, CD 1012 Riley, C 134 Riley, RD 1219 Rimm, DL 941 Rinaldi, S 1817 Risbridger, GP 1784 Robb, SD 684 Roberts, LR 1385 Robinson, D 527 Robsahm, TE 450 Rocca, A 28 Röcken, C 352 Roddam, AW 1817 Rodriguez, JA 145 Rogers, HA 1292 Rogers, M 274 Rohwer, N 772 Rolff, J 950 Ronderos, M 1184 Roos, WP 322 Ros, MM 1817 Rose, P 1852 Rosenberg, L 1486 Ross, JA 63 Rosset, A 1771 Rossi, D 881 Rossi, M 558 Rossing, MA 412, 993 Rosthoej, S 853, 1212 Routledge, JA 1558 Roxburgh, CSD 701 Roy, S 1128 Royer, R 421 Rubagotti, A 1608 Rubeca, T 259 Ruben, SM 1367 Rumjahn, SM 1465 Ruosaari, S 1336 Ruppert, A-M 985 Russo, A 840 Ryan, MC 1367

Sabharwal, A 1250 Sabourin, J-C 1330 Sacerdote, C 1817 Sadanandam, A 1638 Sadeghi, S 1514 Sadetzki, S 1021 Sadhegi, S 795 Saeki, N 389 Sahu, RP 1425 Saijo, N 464, 870 Sainsbury, R 959 Saito, Y 870 Sakamoto, H 389 Salazar, J 1368 Saldanha, JD 1530 Saletti, P 1087 Salmaggi, A 28 Salmenkivi, K 1336 Salmon, I 1444 Salto-Tellez, M 676 Salvat, J 601 Salvi, S 1608 Samali, A 1415 Samoli, E 1794 Sánchez, M-J 1817 Sano, T 501 Santarius, T 370 Santos, JR 1777 Santos, L 89, 487 Sasajima, Y 1400 Sasaki, H 389, 1957 Sasaki, M 1320 Sasako, M 153, 1937 Sasazuki, S 1812 Sauerbrei, W 1219 Saunders, MI 644 Sautter-Bihl, M-L 1680 Savage, P 1061 Sawada, J 870 Sawada, M 1400 Sawyer, MB 1581 Sbarbati, A 1575 Scardino, P 888 Schädler, S 656 Schaedel, D 1731 Schaeffner, E 360 Schairer, C 817 Schatzkin, A 551 Schedvins, K 1303 Schepeler, T 511 Schildkraut, JM 412 Schlag, PM 352, 772 Schleiermacher, G 1471 Schleutker, J 1846 Schmezer, P 1680 Schmidt, CW 322 Schmidt, LS 185 Schmid-Alliana, A 1755 Schmid-Antomarchi, H 1755 Schmiegelow, K 185 Schmitt, F 487 Schmitt-Graeff, A 1949 Schoenleber, SJ 1385 Schoffski, P 44 Schroeder, H 853, 1212 Schueller, H 291 Schüz, J 185 Schwarz, R 908 Schwarz, S 623 Scoazec, JY 1755 Scorilas, A 1659 Scott, J 421 Searle, PF 1903 Seckl, MJ 979 Sedano, L 1368 Seeger, RC 1627 Segara, D 405 Sehgal, A 167 Sehouli, J 1731 Seimiya, H 1369 Sekine, I 1037 Sekyere, E 96 Seliger, B 656 Sellers, TA 412 Selwood, DL 1209 Semba, S 1438 Separovic, D 626 Serkova, NJ 923 Serraino, D 840 Serrano, D 932 Setälä, M 1315 Setoguchi, T 1957 Sewell, R 1746 Shaaban, AM 1393 Shah, K 1191 Shah, M 1806 Shah, N 676 Shah, R 1687 Shalaby, A 1406 Shand, N 315 Shanley, S 376 Sharma, R 707 Sheahan, K 381 Shehadeh, NJ 1379 Sheridan, J 381 Sherris, D 932 Shi, J-F 532 Shibata, D 834 Shibata, T 464, 1257 Shibayama, M 494 Shiels, MS 799 Shimizu, C 50 Shimoda, T 389 Shimura, T 1320 Shindoh, M 1943 Shinomiya, K 747 Shirane, M 764 Shu, X 829, 1499 Sier, CF 1617 Siersema, PD 70 Silva, RR 881 Silver, A 1666 Simon, R 511 Singer, S 908 Singh, RK 1638 Singh, S 1638 Sinnatamby, R 1873 Sinnett, HD 160 Skog, J 1603 Skoglund Lundin, J 1674 Slade, MJ 160, 1879 Sleijfer, DTh 1861 Slimani, N 1817 Smadja-Joffe, F 918 Smalley, KSM 431 Smit, AJ 1861 Smit, EF 145 Smit, VTHBM 494 Smith, D 246 Smith, H 24 Smith, IE 305 Smith, L 1393 Smith, LM 1367 Smith, MJ 1452 Smith, P 1687 Smith, S 96 Sobo, M 106 Soerjomataram, I 77 Solano, S 1111 Solaymani-Dodaran, M 178 Sonabend, AM 1154 Song, B 1674 Song, H 412, 993 Song, HS 298 Sooman, L 334 Soong, R 676 Soosaipillai, A 1659 Sørensen, FB 511 Sørensen, HT 200, 1503 Spanevello, MD 1095 Speight, PM 1128 Speirs, V 1393 Speleman, F 1471 Spencer, EA 1817 Sperl, W 1434 Spina, B 1608 Spitale, A 1087 Spitz, R 1471 Spurdle, AB 412 Srivastava, SK 1425 Stacker, SA 1784 Stahl, M 44 Stalpers, LJA 913 Starling, N 1725 Stassen, LPS 70 Stattin, P 1817 Stavridi, F 1725 Stebbing, J 160, 1061 Steele, N 758 Steinbach, S 44 Steinberg, SM 145 Steinberg, W 1303 Stenman, U-H 1540 Stern, DF 941 Stevens, C 370 Steyerberg, EW 70 Stickney, D 1068 Stier, S 291 Stievano, L 1549 Stirling, JJ 644 Stocken, DD 246 Stoeber, K 959 Stone, IJ 113 Stott, B 1061 Stracci, F 840 Stratton, MR 370 Strickland, AH 37 Strommer, S 1739 Su, J 633 Suárez, LR 1817 Suarez, N 1111 Suchy, J 1508 Sugihara, K 782 Sugimoto, T 399 Sugiyama, E 870 Suligoi, B 840 Sullivan, TJ 1755 Sullivan-Halley, J 524, 1483 Sultana, R 501 Sun, C-A 578 Sun, P 421, 1508 Sundquist, J 829, 1499 Sundquist, K 829, 1499 Sundström, J 874 Sung, JJY 663 Sung, L 82, 1026 Sutherland, MK 1367 Sutherland, RL 123, 405 Sutton, A 436 Suvitie, P 1315 Swanton, C 1517 Sweep, FC 979 Swerdlow, AJ 1832 Swindell, R 1558 Swords, R 1523 Syrjänen, K 874 Szegezdi, E 1415 Szwiec, M 1508

Tagliabue, G 840 Takagi, Y 782 Takano, E 405 Takeda, Y 1647 Takekoshi, S 764 Takemasa, I 1647 Talbot, IC 1666 Talieri, M 1659 Talvinen, K 874 Talwalkar, JA 1385 Tammela, TLJ 1846 Tamminen, A 1055 Tamura, K 50 Tamura, T 464, 1037 Tan, BH 63 Tan, EY 405 Tanai, C 1037 Tanaka, M 1957 Tanaka, S 399, 1943 Tanaka, Y 181 Tang, HL 118 Tang, HM 118 Tani, T 389 Tanida, S 1320 Taniguchi, H 389 Tao, Q 663 Tarca, AL 626 Tarp, M 1746 Taylor, NJ 644 Taylor-Papadimitriou, J 1746 Teall, TJ 1393 Teixeira, MR 487 Tekkis, PP 1666 Terent, A 811 Terry, KL 412 Tessandori, R 840 Testori, A 28 Thai, J 1465 Thomas, B 89 Thomas, CM 979 Thomas, J 1725 Thomas, NPB 1245 Thomas, RJ 598 Thomas, W 96 Thompson, AM 1687 Thompson, J 1725 Thomsen, HF 200 Thomsen, RW 1503 Thomson, D 1250 Thorncroft, M 1250 Thorsen, K 511 Thunnissen, FBJM 145 Tian, L 663 Tichet, J 1230 Tilanus, HW 70 Tinge, B 334 Tirabosco, R 1406 Titus-Ernstoff, L 412 Tjalma, WAA 971 Togashi, Y 1135 Toi, M 281 Tokuda, Y 764 Tong, L 1912 Tookman, A 274 Torresi, U 881 Torres-Rendon, A 1128 Tosetto, M 381 Toso, S 1549 Tosti, G 28 Totsuka, Y 1943 Tougeron, D 1330 Tovey, SM 680 Tran, KTC 70 Trauger, R 1068 Travis, RC 1817 Trenerry, MK 134 Tretli, S 450 Triaridis, S 670 Trichopoulos, D 1794, 1817 Trichopoulou, A 1817 Trinh, XB 971 Troisi, R 1794 Trojan, J 1032 Truini, M 1608 Tsau, H-S 563 Tsuchiya, K 399 Tsuda, H 1400 Tsugane, S 181, 1812 Tsuji, M 848 Tsuruo, T 1369 Tsutsumida, H 344 Tucker, K 583 Tudur Smith, C 246 Tuech, J-J 1330 Tumino, R 840, 1817 Tunon de Lara, C 1048 Turley, H 405, 1666 Tusquets, I 442 Tutill, HJ 476 Tymrakiewitcz, M 426

Ubbink, DT 913 Ueno, H 870 Ulasov, IV 1154 Umemura, S 764 Umeshita, K 1647 Untch, M 590 Urquhart, R 56 Ursin, G 524 Usadel, H 360 Uysal-Onganer, P 1165

Vallee, JP 1230 van Alphen, RJ 228 van Ballegooijen, M 1103, 1240 van Dam, PA 971, 1277 van de Poll-Franse, LV 901 van de Velde, CJH 494 Van Den Berg, DJ 412, 993 van den Berg, MP 1861 Van den Eynden, G 971 Van der Auwera, I 971, 1277 van der Beek, EM 311, 713, 1014, 1211 van der Gaast, A 70 van der Togt, ACM 1103 van der Valk, H 1103 van Doorn, L-J 194 van Haaften, G 370 van Helvoort, A 311, 713, 1014, 1211 van Krieken, JHJM 266 Van Laere, SJ 971, 1277 Van Laethem, J-L 1444 van Leerdam, ME 1103 Van Marck, EA 1277 van Nes, JGH 494 van Norren, K 311, 713, 1014, 1211 Van Orden, KL 1367 van Rooijen, N 113 van Tuijl, S 311, 1014 van Vuuren, AJ 1103 van Weerden, WM 13 Vandrovcova, J 1674 Vanhala, E 1336 Varbanova, M 360 Varney, ML 1638 Vasile, E 1720 Vass, SO 1903 Vejborg, I 1205 Velentzis, LS 1492 Venot, C 366 Vercelli, M 840 Vercoutter-Edouart, A-S 1746 Verghese, ET 1393 Vermeulen, PB 971, 1277 Verrill, MW 684 Verschuur, EML 70 Verspaget, HW 511 Vieira, J 487 Vierkant, RA 412 Vieth, M 352, 772 Vincent, TJ 213 Virgo, JD 376 Visioli, CB 259 Vitarelli, S 840 Vitonis, AF 412 Vodermaier, A 590 Voegeli, A-C 985 von Fournier, D 1680

Wabinga, HR 799 Wacheck, V 1739 Wacher, J 853, 1212 Wada, H 1647 Wada, T 1320 Wailoo, A 436 Wakita, D 1135 Wall, NR 1073 Wallis, MG 1873 Wallström, P 1799 Walsh, G 305 Wanders, A 334 Wanek, T 1739 Wang, EL 501 Wang, H 1784 Wang, H-J 89 Wang, J 563, 1912 Wang, JH 1452, 1589 Wang, L-M 381 Wang, X-H 1347 Wang, Y 376, 1912 Ward, AC 134 Ward, B 160 Wardega, P 334 Wardley, AM 684 Wark, PA 1824 Watanabe, K 464 Watson, AJ 1245, 1250 Watson, E 1852 Watters, JM 56 Waxman, J 1165 Webb, A 571 Webb, PM 412, 795, 1514 Weber, B 601 Wedrén, S 1358 Weller, D 1852 Weller, RE 1926 Wenz, F 1680 Werelius, B 1674 Werner, M 360 Werzowa, J 1739 West, D 524 West, J 178 White, S 1068 Whiteman, DC 795, 1514 Whittemore, AS 412, 993 Widmark, A 1603 Wiedenmann, B 352, 772 Wiemer, EAC 228 Wigmore, SJ 63 Wihlm, J-M 985 Wikman, H 1336 Wilkens, LR 412 Wilkinson, C 1852 Wilkinson, R 426 Willcock, T 206 Williams, ED 1784 Williams, GH 959 Williams, M 598 Williams, R 274 Wilschut, JA 1103 Wilson, JI 644 Wilson, M 723 Wilson, WR 1903 Winterhoff, B 89 Wirfält, E 1799 Wishart, GC 1873 Wokolowczyk, D 1508 Wolin, KY 611 Wong, AHY 663 Woodside, JV 1492 Woodward, N 617 Wotherspoon, A 1725 Wozniak, E 993 Wu, AH 412, 993, 1483 Wu, L 1912 Wu, M-H 578 Wu, R-F 532 Wu, X 334

Xie, X 747 Xu, A 1912 Xu, B 1794 Xu, Y 633 Xue, H 1581 Xynopoulos, D 1659

Yagüe, E 1879 Yagyu, S 399 Yamada, H 782 Yamada, N 344 Yamaji, T 1812 Yamamoto, N 1037 Yamamoto, S 1037 Yamanaka, Y 50 Yan, M 405 Yan, Y 611 Yang, G 1912 Yang, H-K 732 Yanofsky, R 82 Ychou, M 1330 Yeh, CN 1765 Yen, AM-F 563 Yi, SY 894 Ying, J 663 Yokdang, N 1465 Yokobori, T 1937 Yokoyama, A 464 Yokozaki, H 1438 Yonemori, K 50 Yonezawa, S 344 Yoshida, T 389, 870 Yoshikawa, D 1257 Yoshioka, S 1647 Young, A 864 Young, J 959 Yu, C-P 578 Yu, J 663, 1842 Yu, J-C 578 Yu, VPCC 376 Yuan, J-M 834 Yuen, KL 118 Yunokawa, M 50

Zadeh, G 1020 Zaffaroni, N 739 Zahl, P-H 219 Zaidi, SAA 160 Zalcberg, JR 37 Zambon, P 840 Zanetti, R 840 Zanke, BW 1966 Zappa, M 259 Zelada-Hedman, M 1674 Zeng, L 747 Zeps, N 676 Zerillo, C 941 Zhang, W 206, 213 Zhang, W-H 532 Zhang, Y-Z 532 Zhao, G 1912 Zhao, Y 1912 Zheng, Y 1659 Zhou, X 1674 Zhu, L 1912 Zhu, Y 747 Zijlmans, HJ 1617 Zimmermann, FA 1434 Ziogas, A 412 Zoellner, U 315 Zorzi, M 259 Zsebedics, M 1739

